# Protocol for the isolation and characterization of mouse alveolar bone marrow hematopoietic stem cells

**DOI:** 10.1016/j.xpro.2025.103875

**Published:** 2025-06-05

**Authors:** Kouta Niizuma, Satoru Morikawa, Eric Gars, Alyssa H. Chang, Adam C. Wilkinson, Hiromitsu Nakauchi, Ryo Yamamoto

**Affiliations:** 1 Institute for Stem Cell Biology and Regenerative Medicine, Stanford University School of Medicine, Stanford, CA 94305, USA; 2 Department of Genetics, Stanford University School of Medicine, Stanford, CA 94305, USA; 3 Division of Stem Cell Therapy, Distinguished Professor Unit, Institute of Medical Science, The University of Tokyo, Tokyo, Japan; 4 Department of Dentistry and Oral Surgery, Keio University School of Medicine, Tokyo, Japan; 5 MRC Weatherall Institute of Molecular Medicine, Radcliffe Department of Medicine, University of Oxford, Oxford, UK; 6 Institute for the Advanced Study of Human Biology (WPI-ASHBi), Kyoto University, Kyoto 606-8501, Japan

**Keywords:** cell Biology, cell culture, cell isolation, flow cytometry, Model Organisms, stem cells

## Abstract

Here, we present a protocol for isolating and characterizing hematopoietic stem cells (HSCs) from mouse alveolar bone marrow. We describe steps for combining anatomical dissection of the mandibular alveolar region with flow cytometry analysis for identifying HSCs. The protocol enables the isolation of HSCs from a specific bone marrow niche harboring an enriched HSC population.

For complete details on the use and execution of this protocol, please refer to Niizuma et al.^1^

## Highlights

• Protocol for isolating hematopoietic stem cells from mouse alveolar bone marrow
• Instructions for anatomical dissection of the mandibular alveolar region
• Procedure for identifying HSCs from this enriched niche using flow cytometry

## Abstract (editor highlights)

Publisher’s note: Undertaking any experimental protocol requires adherence to local institutional guidelines for laboratory safety and ethics.

## Graphical abstract

**Figure:**
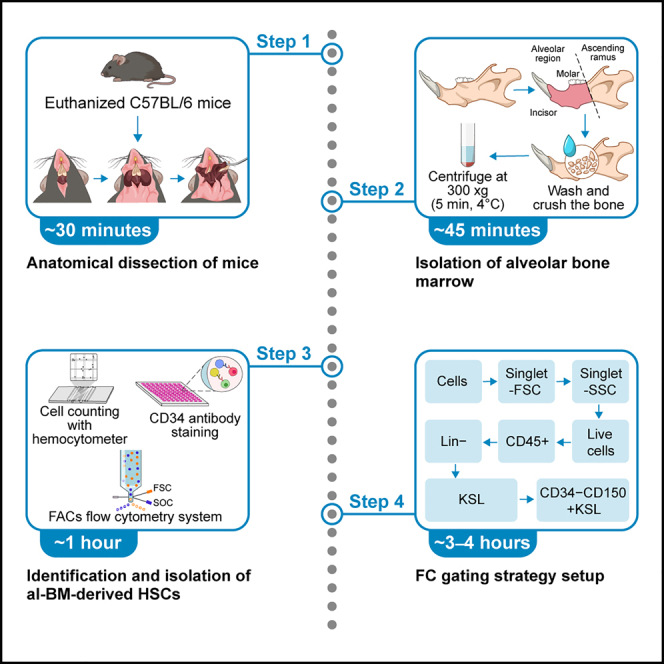

## Before you begin

### Background

Hematopoietic stem cells (HSCs), located at the apex of the hematopoietic hierarchy, are essential for maintaining blood homeostasis. Recent studies have demonstrated that self-renewal and multipotency are mutually exclusive properties of HSCs and that distinct subpopulations of HSCs exhibit lineage biases,^2^ such as those found in bone marrow niches, including the perivascular localization of Hoxb5+HSCs.^3^ While studies have traditionally focused on HSCs derived from the bone marrow (BM) in the femur, tibia, and pelvis, substantial site-specific differences in HSC properties have been observed across various skeletal regions in mice. These site-specific HSCs can be valuable for targeted therapeutic applications, transplantation studies, and understanding the fundamental mechanisms of tissue-specific hematopoiesis.

Our recent study reported that the alveolar bone marrow (al-BM) within the mouse mandible harbors the highest concentration of immunophenotypic HSCs among different skeletal sites.^1^ The engraftment of al-BM HSCs is superior to those from the femur, tibia, and pelvic BM, as shown using transplantation assays. Additionally, the immune microenvironment of al-BM has been demonstrated from that of other skeletal sites, potentially contributing to the unique properties of al-BM HSCs.^4^ These findings greatly improve the understanding of regional hematopoiesis and reveal a previously underexplored BM niche with distinct biological characteristics.

To further investigate this specialized HSC population, we present a protocol for efficiently isolating and characterizing HSCs from al-BM. This protocol combines precise anatomical dissection and optimized flow cytometry (FC) to enable reproducible studies on al-BM HSCs. Using this protocol, researchers can gain new insights into the unique microenvironment and functional properties of HSCs in alveolar bone, contributing to hematopoiesis research and regenerative medicine.

This protocol is based on established methodologies for ex vivo HSC expansion^5^ and characterization techniques adapted from standardized protocols.^6^ This technique ensures reproducible collection and analysis, facilitating comparative studies with long bones.^1^

### Experimental animals

All animal experiments were conducted in compliance with the regulations of the respective institutions. In this protocol, 8–12-week-old C57BL/6 (C57BL/6-CD45.2) male mice purchased from the Jackson Laboratory (Cat. no. 000664) were used.

### Institutional permissions

Animal experiments were performed with the approval of the Stanford University Institutional Animal Care and Use Committee (Protocols #33113 and #33171).

**CRITICAL:** Experimenters must obtain the necessary approval according to the regulations of their respective institutions before beginning the animal experiments.

### Preparation for experiment

**Timing: 1–2 h**

1. Prepare and filter all required buffers (see materials and equipment section for recipes).
2. Check expiration dates and optimal dilutions for all antibodies (see key resources table).
3. Ensure the availability of all required materials and equipment (see key resources table).
4. Prepare necessary documentation (e.g., experiment worksheets, data collection templates, analysis protocols).
5. Label all necessary tubes and plates clearly.
6. Warm up the flow cytometer lasers for at least 30 min before use.
7. Run cytometer performance validation using CS&T beads and check fluidics system.
8. Prepare compensation controls (single-stained and unstained).
9. Test Propidium Iodide (PI) staining on control samples.
10. Verify the proper functioning of the cell sorter, including drop delay calibration.

**CRITICAL:** All reagents and equipment must be prepared before starting cell isolation to minimize the time between tissue collection and analysis.

## Key resources table

**Table:** 

REAGENT or RESOURCE	SOURCE	IDENTIFIER
**Antibodies**		

Biotin anti-mouse CD4 (clone RM4-5) (1:2,800)	eBioscience	Cat# 13-0042-85; RRID: AB_466330
Biotin anti-mouse CD8 (clone 53-6.7) (1:2,800)	eBioscience	Cat# 13-0081-86; RRID: AB_466348
Biotin anti-mouse CD45R/B220 (clone RA3-6B2) (1:1,400)	eBioscience	Cat# 13-0452-85; RRID: AB_466450
Biotin anti-mouse TER-119 (clone TER-119) (1:700)	eBioscience	Cat# 13-5921-85; RRID: AB_466798
Biotin anti-mouse Gr-1/Ly-6G (clone RB6-8C5) (1:700)	eBioscience	Cat# 13-5931-85; RRID: AB_466801
Biotin anti-mouse CD127 (clone A7R34) (1:1,400)	eBioscience	Cat# 13-1271-85; RRID: AB_466589
Streptavidin APC-eFluor 780 (1:400)	eBioscience	Cat# 47-4317-82; RRID: AB_10366688
APC anti-mouse c-Kit (clone 2B8) (1:400)	eBioscience	Cat# 17-1171-83; RRID: AB_469431
PE anti-mouse Sca-1/Ly-6A-E (clone D7) (1:400)	BioLegend	Cat# 122508; RRID: AB_756193
PE/Cy7 anti-mouse CD150 (clone TC15-12F12.2) (1:400)	BioLegend	Cat# 115914; RRID: AB_439797
FITC anti-mouse CD34 (clone RAM34) (1:100)	eBioscience	Cat# 11-0341-85; RRID: AB_465022
Brilliant Violet 510 anti-mouse CD45.2 (clone 104) (1:400)	BioLegend	Cat# 109837; RRID: AB_2561393

**Chemicals, peptides, and recombinant proteins**		

PI solution	BioLegend	421301
Sterile phosphate-buffered saline (PBS)	Gibco	10010–023

**Experimental models: Organisms/strains**		

*Mus musculus* | C57BL/6 [CD45.2] | 8–12 weeks old | male and female (yield data in expected outcomes derived from both sexes)	Jackson Laboratory	000664

**Other**		

FACSAria II cell sorter	BD Biosciences	N/A
Dissecting microscope	Leica	M165 FC
CS&T Beads	BD Biosciences	656504
Sterile surgical scissors	Fine Science Tools	91460-11
Fine forceps	Fine Science Tools	*91100*-*12*
Mortar and pestle	Fisher Scientific	FB970K
70-μm cell strainer	Falcon	352350
15 mL conical tubes	Falcon / Fisher Scientific	352096

## Materials and equipment

### Reagent preparation

**Table:** FACS buffer preparation

Reagent	Final concentration	Amount
PBS	N/A	499.5 mL
PI	0.1%	0.5 mL
Total	N/A	500 mL

Store FACS buffer at 4°C protected from light for up to 1 month.

## Step-by-step method details

### Mouse alveolar BM isolation and cell preparation

**Timing: 1 h**

This section describes the surgical isolation and preparation of a single-cell suspension of al-BM from mouse mandible. This protocol differs from the standard long BM isolation protocol because of the anatomical complexity of the mandible. Moreover, this procedure can be performed for femur, tibia, and pelvic, BM isolation if comparative analysis is required (Niizuma et al., 2024).^1^

1. Prepare surgical tools and materials:
  a. Place sterile surgical tools (scissors, fine forceps and fine scissors) in a sterile field.
  b. Prepare ice-cold PBS (Gibco, Cat# 10010-023) in a sterile tube.
  c. Set up a sterile work area with paper towels.
2. Expose the mandible:
  a. Euthanize 8–12-week-old C57BL/6 mice using CO_2_ inhalation followed by cervical dislocation according to the institutional guidelines.
  b. Create a skin incision along the lateral side of the face to fully expose the masticatory muscles.
  c. Carefully remove the skin from the lower jaw to expose the underlying muscle structures.
  d. Open the mouth to improve access to the mandibular region.
3. Dissect the Mandible:
  a. Identify and carefully detach the masticatory muscles from the mandible.
  b. Remove the major muscle groups using surgical tools.
  c. Using Kimwipes, gently remove the residual muscle tissue that could not be dissected using forceps.
  d. Locate the temporomandibular joint and carefully detach it to separate the mandible from the skull. Notably, the condylar process serves as a key anatomical landmark for precise dissection.
  e. Extract the intact mandible while ensuring minimum damage to the alveolar bone.

**CRITICAL:** Constantly maintain samples at an ice-cold temperature (0°C–4°C) by keeping the tissues on ice to preserve cell viability.

**Figure 1:**
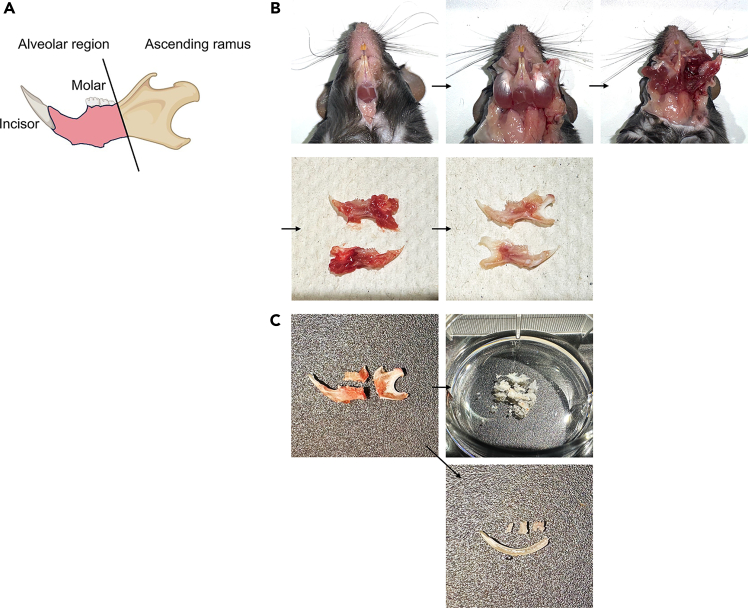
Procedure for isolating mouse alveolar bone marrow (A) Anatomical landmarks for alveolar bone isolation, showing the mandible with an alveolar region (pink) and an ascending ramus. (B) The step-by-step dissection process showing the removal of masticatory muscles and isolation of the alveolar bone. (C) The dissected alveolar bone and teeth.

4. Isolate the alveolar bone:
  a. Identify the alveolar region between the molar and incisor areas (Figures 1A–1C).
  b. Carefully dissect the alveolar bone using fine scissors.^1^
  c. Avoid damage to the tooth roots and dental pulp tissue.
5. Processing the BM:
  a. Wash the isolated alveolar bone with ice-cold PBS (Gibco, Cat# 10010-023).
  b. Crush the bone tissue using a mortar and pestle.
  c. Filter the cell suspension through a 70-μm cell strainer (Falcon, Cat# 352350).
  d. Centrifuge at 300 ×g for 5 min at 4°C.
  e. Resuspend the cell pellet in PBS.

***Note:*** Unlike other BM isolation protocols, this isolation technique does not include a red blood cell (RBC) lysis step, as the HSCs obtained were of sufficient purity for downstream applications. Moreover, RBC lysis can sometimes negatively impact the viability of sensitive stem/progenitor cells.

**Pause point:** The cell suspension can be kept on ice for up to 1 h before proceeding to the next step.

**Figure 2:**
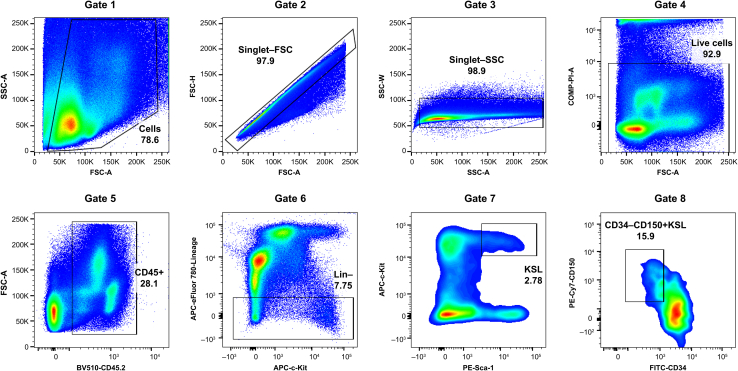
Flow cytometric analysis and gating strategy for identifying hematopoietic stem cells Representative flow cytometry plots showing the sequential gating strategy for identifying HSCs in the alveolar bone marrow. (Gate 1) FSC-A vs. SSC-A to identify cells. (Gate 2) Singlet-FSC discrimination. (Gate 3) Singlet-SSC discrimination. (Gate 4) Live cell gating. (Gate 5) CD45+ hematopoietic cells. (Gate 6) Lineage-negative cells. (Gate 7) KSL (c-Kit+Sca-1+Lin−) cells. (Gate 8) CD34−CD150+KSL HSCs. The numbers indicate the percentage of cells in each gate. The plots shown are representative of data obtained from male mice.

### Flow cytometric identification and isolation of al-BM-derived HSCs

**Timing: 3–4 h**

This step describes the detailed procedure for identifying and isolating al-BM-derived HSCs using multicolor FC. This protocol can be used to identify long-term HSCs based on their immunophenotype (CD34−CD150+KSL).

6. Sample preparation and antibody staining:
  a. Filter the cell suspension through a 40-μm cell strainer to remove any remaining bone fragments.
  b. Count cells using a hemocytometer and adjust to 1–5 × 10^6^ cells/mL in cold FACS buffer.
  c. Transfer cells to 5-mL FACS tubes.
  d. Add lineage cocktail antibodies (3 μL per 1 × 10^7^ cells).
  e. Prepare the lineage cocktail master mix:
    ***Note:*** Prepare the lineage cocktail master mix (Table 1) in advance and store it at 4°C, protected from light for up to 3 months. We typically use 3 μL of the master mix for every 1 × 10⁷ cells.
    **CRITICAL:** Titrate the volume of each antibody before use to optimize staining.

**Table 1:** Lineage cocktail master mix preparation

Reagent	Concentration	Volume (μL)
Biotin-Gr1/Ly-6G	0.5 mg/ml	100
Biotin-Ter119	0.5 mg/ml	100
Biotin-CD4	0.5 mg/ml	25
Biotin-CD8	0.5 mg/ml	25
Biotin-CD45R/B220	0.5 mg/ml	50
Biotin-CD127	0.5 mg/ml	50
Sterile PBS	N/A	350
Total	N/A	700

Components and volumes for preparing the lineage antibody cocktail used for marking and excluding differentiated hematopoietic cells in flow cytometry analysis.
  f. Incubate for 30 min on ice in the dark.
  g. Wash with 5 mL cold PBS and centrifuge at 440 ×g for 5 min at 4°C.
  h. Add fluorochrome-conjugated antibodies at predetermined optimal concentrations:
    i. Streptavidin-APC-eFluor 780 (1:400).
    ii. APC-c-Kit (1:400).
    iii. PE-Sca-1 (1:400).
    iv. PE/Cy7-CD150 (1:400).
    v. FITC-CD34 (1:100).
    vi. BV510-CD45.2 (1:400).
  i. Incubate on ice in the dark for 90 min.
    **CRITICAL:** The 90-min incubation is essential for optimal CD34 staining.
7. FC setup and quality control:
  a. Turn on the flow cytometer and allow the lasers to warm up (minimum 30 min).
  b. Run CS&T beads for performance validation.
  c. Setup compensation using single-stained controls.
    i. Use cells for all markers except rare antigens.
    ii. Set up compensation using single-stained dells for all markers.
  d. Verify the compensation matrix accuracy.
  e. Set FSC and SSC voltages using unstained cells.
  f. Confirm the proper separation of positive and negative populations using Fluorescence Minus One (FMO) controls to accurately set gating boundaries that are affected by spectral overlap/spread.
8. Sample acquisition and analysis:
  a. Add PI, a DNA-binding fluorescent dye that cannot penetrate intact cell membranes and is used to identify dead cells, at a final concentration of 0.1% immediately before the analysis.
  b. Set up the sequential gating strategy (Figure 2).
    i. FSC/SSC to identify cells (typical setting: FSC-A vs. SSC-A).
    ii. Doublet discrimination using FSC-A vs. FSC-H and SSC-A vs. SSC-W.
    iii. Live cells (PI-negative).
    iv. CD45+ hematopoietic cells.
    v. Lineage-negative cells.
    vi. c-Kit+Sca-1+ (KSL) cells.
    vii. CD34−CD150+ HSCs.
  c. Record cells.

**CRITICAL:** Maintain the acquisition rate below 5,000 events/s to maintain data quality.

***Note:*** The samples are maintained on ice throughout the procedure.

**Pause point:** The stained samples can be kept on ice in FACS buffer for up to 1 h before analysis.

## Expected outcomes

Following this protocol, researchers should be able to isolate and analyze HSCs from the mouse al-BM successfully. A typical yield of 4–12 × 10^5^ BM cells per alveolar bone from a single mouse represents the successful isolation of the alveolar BM and is considered optimal for downstream applications.

When comparing male and female mice, the typical yield from male mice is 8.47 ± 3.71 × 10^5^ cells per alveolar bone, while the approximate yield from female mice is 7.6 ± 2.69 × 10^5^ cells. Comparatively, the yield from the femur, tibia, and pelvic BM (ftp-BM) was considerably higher. 1.2 ± 0.23 × 10^8^ cells and 1.12 ± 0.34 × 10^8^ cells were obtained from male and female mice, respectively, per combined ftp-BM.

The cell suspension appears slightly pink and contains small, white bone fragments. Cell viability exceeded 90%, as assessed by the PI staining. The clean removal of the alveolar bone region without contamination with the dental pulp tissue indicates successful isolation. Depending on the sorting strategy, the yield might be lower for the HSC-enriched fractions.

The analysis of properly prepared samples should reveal distinct cell populations with a clear separation between the positive and negative fractions. Representative FC plots displaying the sequential gating strategy are shown in Figure 2. The increased frequency of HSCs in the al-BM compared with the femur/tibial bone marrow (∼2-fold higher) is an internal validation of successful isolation. Poor separation of cell populations or unusually low staining frequencies may indicate suboptimal antibody staining or sample processing.

Quality control parameters for FC analysis are critical for ensuring reliability. FSC/SSC profiles should show a clear separation of cell populations, while compensation controls should show minimal spillover between channels. Effective doublet discrimination should remove >95% of the aggregates and dead cell exclusion should show a clear separation between PI+ and PI− populations. If the cell yields or population frequencies deviate markedly from the expected values, refer to the troubleshooting section for potential causes and solutions.

## Quantification and statistical analysis

FC data analysis was performed using FlowJo software (BD Biosciences). The sequential gating strategy for HSC identification followed standardized steps (Figure 2), with the quantification of cell populations at each stage. Initial quality control gates included cell identification (FSC-A vs. SSC-A, Gate 1), doublet exclusion (FSC-H vs. FSC-A, Gate 2; SSC-H vs. SSC-W, Gate 3), and live/dead discrimination (PI-negative selection, Gate 4). HSC identification gates were CD45+ cells (Gate 5), lineage-negative cells (Gate 6), KSL (c-Kit+Sca-1+Lin−) cells (Gate 7), and CD34−CD150+KSL HSCs (Gate 8). Population frequency was expressed as a percentage of the parent gate, and absolute cell numbers were calculated using the total cell count. For statistical considerations, at least three independent experiments should be performed. Data should be presented as mean ± standard error of the mean (SEM), and appropriate statistical tests should be used for comparisons between groups.

## Limitations

This protocol has several technical and biological limitations that should be considered. The trabecular structure of the alveolar bone makes it impossible to isolate pure BM without mechanical processing.

Because of this structural complexity, the absolute HSC numbers per bone volume cannot be accurately quantified. Additionally, mechanical processing may affect the expression of cell surface markers and, consequently, the immunophenotypic analysis of HSCs.

From a technical perspective, surgical expertise is required for precise alveolar bone dissection, which might be challenging for new researchers. The small size of the alveolar bone tissue limits the experimental scale and total cell yield compared to long bones. Moreover, processing time constraints are critical for maintaining cell viability, requiring efficient workflow from tissue collection to analysis.

The analysis methodology also has inherent limitations. FC analysis is limited to surface markers, which may not completely capture the functional heterogeneity of HSCs. Cell populations are defined by immunophenotype rather than functional assays may not always correlate with in vivo HSC activity.

Furthermore, compensation complexity increases with multiple fluorochromes, potentially affecting the resolution of rare populations.

From a biological perspective, this protocol was optimized based on 8–12-week-old C57BL/6 mice, and strain- and age-dependent variations may affect the results when applied to different mouse models. Individual variations in bone structure may impact cell yield and HSC frequency, necessitating appropriate statistical approaches when comparing different experimental groups.

## Troubleshooting

### Problem 1

Low cell yield from alveolar bone (Step 1e). This issue can arise from several factors, including incorrect identification of the alveolar region during dissection, insufficient mechanical processing when crushing the bone tissue, cell loss during the filtration step, or poor cell viability due to temperature fluctuations or inadequate buffer conditions.

### Potential solution

To ensure correct identification, review the anatomical landmarks provided in Figures 1A–1C. Ensure the bone tissues are thoroughly crushed using a mortar and pestle while maintaining samples at ice-cold temperature (0°C–4°C) throughout the procedure. Additionally, verify the pH and osmolarity of the buffer before use.

### Problem 2

Poor separation of cell populations in FC (Step 2). Poor separation may be caused by suboptimal antibody staining (incorrect concentration or incubation time), improper fluorescence compensation, sample aggregation, or a high level of dead cell contamination.

### Potential solution

Verify that optimal antibody concentrations and incubation times were used according to the protocols (Step 2a). Prepare fresh compensation controls for each experiment using single-stained cells (Step 2b). Ensure samples are filtered immediately before analysis to remove aggregates. Add the PI solution just before acquisition to accurately exclude dead cells.

### Problem 3

A high proportion of dead cells (Step 1, Step 2a). Excessive number of dead cells can result from extended processing time (especially during isolation), the application of excessive mechanical force during bone crushing, large temperature fluctuations, or the use of contaminated buffers.

### Potential solution

Aim to complete isolation procedure (Step 1) within approximately 1 h. Employ gentle crushing technique. Maintain consistent cold temperature (0°C–4°C) for all samples and regents. Always prepare fresh, sterile buffers for each experiment.

### Problem 4

Unclear KSL population (Step 2a, 2b, 2c). Difficulty in resolving the KSL population can be due to insufficient staining (especially for CD34, which requires a 90-min incubation), nonspecific antibody binding, poor separation of lineage−negative cells, or an improper gating strategy.

### Potential solution

Ensure the critical 90-min incubation period for CD34 staining (Step 2a.ix) is strictly followed. Include FMO controls to help set gates accurately (Step 2b.vi). Optimize the concentration of the lineage antibody cocktail if Lin- separation if poor (Step 2a.iv, Table 1). Carefully follow the sequential gating strategy depicted in Figure 2 (Step 2c.ii).

### Problem 5

Low purity of the sorted cells (Post-Step 2/Cell sorting). Low purity after cell sorting may be due to an improper drop delay setting on the sorter, using excessive sort pressure, cell clumping in the sample just before sorting, or an unstable stream during the sort.

### Potential solution

Verify the sorter’s drop delay calibration using appropriate beads (e.g., AccuDrop bead). Maintain a low sort pressure (ideally <30 psi, but refer to sorter specifications). Filter the samples immediately before sorting minimize clumps. Regulatory check the nozzle for any debris or potential damage that could affect stream stability.

## Resource availability

### Lead contact

Further information and requests for resources and reagents should be directed to the lead contact, Ryo Yamamoto (yamamoto.ryo.2c@kyoto-u.ac.jp).

### Technical contact

Technical questions on executing this protocol should be directed to Kouta Niizuma (niizuma@stanford.edu).

### Materials availability

No new reagents were generated in this study.

### Data and code availability

The datasets supporting this protocol have not been deposited in a public repository but are available from the corresponding author upon request. No original code was generated for this study.

## Acknowledgments

We thank the Stanford Stem Cell Institute FACS Core for access to the flow cytometry facilities, Catherine Carswell-Crumpton and Cheng Pan for their advice on the flow cytometric assays, and Satoru Matsunaga for his advice on the mandibular histology analysis.

This work was supported by 10.13039/100000052NIH grants R01DK116944 and R01HL147124 and ONUKI KIKIN (for English language editing and preparation of graphical abstracts). The authors would like to thank Enago (www.enago.jp) for the English language review.

## Author contributions

Conceptualization, K.N., S.M., H.N., and R.Y.; methodology, K.N., S.M., A.C.W., and R.Y.; validation, K.N., S.M., E.G., A.H.C., and R.Y.; formal analysis, K.N., S.M., and R.Y.; investigation, K.N., S.M., and A.H.C.; resources, R.Y. and H.N.; data curation, K.N., S.M., and R.Y.; writing – original draft, K.N. and S.M.; writing – review and editing, R.Y., H.N., A.C.W., and A.H.C.; visualization, K.N., S.M., and R.Y.; supervision, R.Y. and H.N.; project administration, R.Y.; funding acquisition, H.N. and R.Y. K.N. and S.M. contributed equally to this study. All authors have read and agreed to the published version of the manuscript.

## Declaration of interests

H.N. is a co-founder and shareholder of Megakaryon, Corp.; Reprocell, Inc.; Celaid Therapeutics; and Century Therapeutics, Inc. A.C.W. is a scientific advisor for ImmuneBridge. None of these companies were involved in the present work.
